# Medical News and Items

**Published:** 1875-12

**Authors:** 


### Medical News and Items.

Obituary.—Dr. F. W. Headland, author of the well known and much prized work on "The Action of Medicines," died recently in London, at the age of forty-six. He was senior physician to Charing Cross Hospital, and was greatly respected by his professional associates.

Obituary.—Dr. John Hughes Bennett, late Professor Institutes of Medicine in the University of Edinburg, is dead. He was born in 1812, and graduated in 1837. He was one of the great teachers of positive medicine. His work on Clinical Medicine is well known to the profession in America, among whom it has been widely read.

The *Indiana Journal of Medicine*, edited by Dr. Thaddeus M. Stevens and published at Indianapolis, has been consolidated with the *Cincinnati Lancet and Observer*, Dr. Stevens acting as assistant editor of the combined journals. Dr. J. C. Culbertson is of course undisturbed in his position of editor.

Copper in Soda Water.—Upon a chemical examination instituted by the magistrate of Munich, of the soda waters, twenty samples out of thirty were found to contain copper to an amount injurious to health. The source of the copper was thought to be a defective lining with tin of the copper tubes used in the apparatus. The strawberry juice was compounded of common syrup, aniline dye and æthereal oil.

The *Fourth International Medical Congress* was held at Bruxelles on Sept. 19th.

The *Forty-Eighth Convention* of German physicians and naturalists was held at Gratz (Austria), on Sept. 20th to 25th. Hamburg was chosen as the place of meeting for next year.

The Corner Stone of the new Rush Medical College building was laid on the 20th ult. with the solemnity of Masonic forms.

A procession was formed in front of Oriental Hall on La Salle Street, at 11 A. M., made up of the officers of the Grand Lodge of Illinois, the Oriental Consistory, the Chicago Commandery, the St. Bernard Commandery, the College Faculties, and the entire class of Students. This column marched, with music, to the new college site, where the ceremony of the occasion was performed by the officers of the Grand Lodge, assisted by President Freer of the College, and the Masonic bodies in attendance.

This completed, the orator of the occasion, Prof. J. Adams Allen, delivered a most able and interesting address.

In the State of New Hampshire, every physician, surgeon, obstetrician, druggist and druggist's clerk must be provided with a "certificate" granted by legally constituted authority, which certificate shall set forth that the holder thereof is properly qualified to practice his profession. Practicing without this certificate is declared a misdemeanor, and the "fine for the first offense is not less than *fifty dollars* nor more than *two hundred dollars;* for any subsequent offense, not less than *two hundred dollars* nor more than *five hundred dollars ;* which fine may be recovered by an action of debt for the use of any person who shall sue therefor, or by an indictment." Each certificate is recorded in a book kept for that purpose, in the county clerk's office, which book is to be known as the Medical Register of said county. The authority for granting these certificates to physicians, is a "board of censors, consisting of not less than three persons," "appointed by each and every medical society, organized under a charter from the legislature of the State of New Hampshire." This board issues, without license, certificates to physicians holding medical diplomas or certificates of examination from some authorized board. It can revoke certificates or declare any man incompetent to practice, for cause. This act does not apply to physicians who have been in practice five years, in that State.

